# Oxidative Stress and Mitochondrial Damage in Dry Age-Related Macular Degeneration Like *NFE2L2/PGC-1α* *^-/-^* Mouse Model Evoke Complement Component C5a Independent of C3

**DOI:** 10.3390/biology10070622

**Published:** 2021-07-04

**Authors:** Iswariyaraja Sridevi Gurubaran, Hanna Heloterä, Stephen Marry, Ali Koskela, Juha M. T. Hyttinen, Jussi J. Paterno, Arto Urtti, Mei Chen, Heping Xu, Anu Kauppinen, Kai Kaarniranta

**Affiliations:** 1 Department of Ophthalmology, Institute of Clinical Medicine, University of Eastern Finland, 70210 Kuopio, Finland; raja.sridevigurubaran@uef.fi (I.S.G.); ali.koskela@uef.fi (A.K.); juha.hyttinen@uef.fi (J.M.T.H.); jussi.paterno@uef.fi (J.J.P.); 2 Department of Ophthalmology, Roche Oy, 02100 Espoo, Finland; hanna.helotera@roche.com; 3 The Wellcome-Wolfson Institute for Experimental Medicine, School of Medicine, Queens University Belfast, Belfast BT9 7BL, UK; s.marry@qub.ac.uk (S.M.); m.chen@qub.ac.uk (M.C.); heping.xu@qub.ac.uk (H.X.); 4 Faculty of Health Sciences, School of Pharmacy, University of Eastern Finland, 70210 Kuopio, Finland; arto.urtti@uef.fi (A.U.); anu.kauppinen@uef.fi (A.K.); 5 Department of Ophthalmology, University of Eastern Finland and Kuopio University Hospital, 70210 Kuopio, Finland

**Keywords:** aging, oxidative stress, mitochondrial damage, age-related macular degeneration, inflammation, complement system, Toll-like receptors, complement factor H, thrombin, C-reactive protein, receptor for advanced glycation end products

## Abstract

**Simple Summary:**

Age-related macular degeneration (AMD) is an eye disease that results in permanent loss of vision due to degeneration in the central portion of the retina called the macula. Patients with severe visual loss have reduced quality of life and the risk of death is 2.4 times higher than the general population. Currently, there is no treatment to stop or cure dry AMD. Aging-associated chronic oxidative stress and inflammation are known to be involved in AMD pathology. To investigate the molecular mechanism behind the cause and to develop novel therapy, we have created and validated an animal model mimicking clinical features of dry AMD. Here, we show previously unknown thrombin-mediated complement component C5a activation in the degenerative retina without upregulation of C3. Our model might provide insight into AMD progression and help to develop novel therapies.

**Abstract:**

Aging-associated chronic oxidative stress and inflammation are known to be involved in various diseases, e.g., age-related macular degeneration (AMD). Previously, we reported the presence of dry AMD-like signs, such as elevated oxidative stress, dysfunctional mitophagy and the accumulation of detrimental oxidized materials in the retinal pigment epithelial (RPE) cells of nuclear factor erythroid 2-related factor 2, and a peroxisome proliferator-activated receptor gamma coactivator 1-alpha (*NFE2L2/PGC1α*) double knockout (dKO) mouse model. Here, we investigated the dynamics of inflammatory markers in one-year-old *NFE2L2/PGC1α* dKO mice. Immunohistochemical analysis revealed an increase in levels of Toll-like receptors 3 and 9, while those of NOD-like receptor 3 were decreased in *NFE2L2/PGC1α* dKO retinal specimens as compared to wild type animals. Further analysis showed a trend towards an increase in complement component C5a independent of component C3, observed to be tightly regulated by complement factor H. Interestingly, we found that thrombin, a serine protease enzyme, was involved in enhancing the terminal pathway producing C5a, independent of C3. We also detected an increase in primary acute phase C-reactive protein and receptor for advanced glycation end products in *NFE2L2/PGC1α* dKO retina. Our main data show C5 and thrombin upregulation together with decreased C3 levels in this dry AMD-like model. In general, the retina strives to mount an orchestrated inflammatory response while attempting to maintain tissue homeostasis and resolve inflammation.

## 1. Introduction

**Figure 1:**
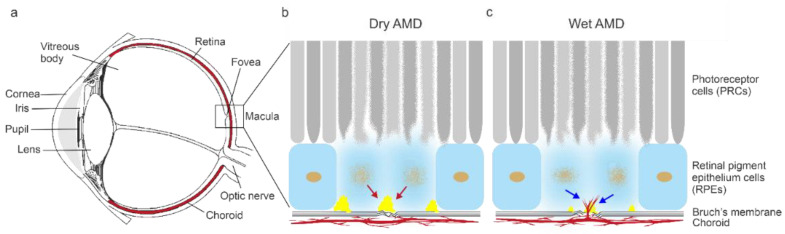
A parasagittal section of the eye (**a**). The clinical hallmarks of dry AMD are an accumulation of extracellular “drusen” deposits (red arrow) and degeneration of RPE cells leading to loss of PRCs (**b**). Wet AMD is characterized by the occurrence of choroidal neovascularization (blue arrow) (**c**).

Age-related macular degeneration (AMD) is the most common cause of vision loss in the elderly. The risk factors of AMD include aging, demographic characteristics, environmental factors, and genetic and metabolic reasons [1,2,3]. The clinical hallmarks of AMD pathophysiology involve the detrimental accumulation of oxidized extracellular materials known as “drusen”, a myriad of inflammation processes, and neovascularization associated with the degeneration of retinal epithelial cells (RPEs) and a loss of photoreceptor cells (PRCs) (Figure 1) [4,5,6].

The combination of aging-related oxidative stress, oxidized proteins and lipid accumulation, and other altered metabolic products, results in a sustained development of a low-grade chronic inflammation via complement activation. These processes are known to trigger secondary effects in many neurodegenerative diseases including AMD [7,8,9,10]. Emerging data from clinical genetic studies have detected gene polymorphic variants among the complement system [11,12,13,14,15].

Some of the features of clinical AMD include a comprehensive expression of various inflammatory associated proteins and receptors [16,17,18]. In general, cells recognize exogenous pathogens and endogenous stimuli via their pathogen-associated (PAMPs) and damage-associated (DAMPs) molecular patterns through the conserved pathogen recognition receptors (PRRs), such as Toll-like (TLRs) and NOD-like receptors (NLRs). TLRs modulate inflammation by transcriptional and post-transcriptional modifications via nuclear factor kappa-B (NF-κB) pathways, whereas NLRs are involved in inflammasome activation and the production of interleukins (IL) [19,20,21].

Low-grade inflammation activates PRRs, such as circulating complement components and pentraxins. The activation of the complement system (CS) triggers a cascade of protease reactions, producing pro-inflammatory mediators. Complement components C3a and C5a are anaphylatoxins that promote inflammation by attracting active mast cells and induce the production of adhesion molecules to increase the permeability of blood vessels. C3a tends to attenuate lipopolysaccharide-induced endotoxemia, primarily activating granulocytes rather than neutrophils, while C5a can recruit neutrophils and monocytes to the site of inflammation and activate these cells in these locations [22,23].

Pentraxins are evolutionarily conserved PRRs that are divided into two groups: short pentraxins, e.g., C-reactive protein (CRP), and long pentraxins, e.g., serum amyloid P (SAP) [21]. Both CRP and SAP are known to be primary acute phase proteins in humans and mice. CRP and SAP are produced in the liver as a systemic response to the presence of several potent pro-inflammatory cytokines. CRP can also be produced locally by many other cell types, including endothelial cells, fibroblasts, adipocytes, and mononuclear phagocytes; these cells respond to various pro-inflammatory signals such as IL-1β, tumor necrosis factor alpha (TNFα), and lipopolysaccharides (LPS) to produce CRP. The presence of agonists can also induce the expression of receptors for advanced glycosylation end-products (RAGE) and activate NF-κB signaling pathways.

Recently, we showed that the global knockout of nuclear factor erythroid 2-related factor 2 and peroxisome proliferator-activated receptor gamma coactivator 1-alpha (*NFE2L2/PGC-1α*), the master regulators of antioxidant production and mitochondrial biogenesis, led to disturbed autophagy, an accumulation of drusen-like deposits, and the infiltration of Iba-1 positive immune cells mimicking clinical features of the dry AMD phenotype [24].

In this study, we show that mitochondrial-derived oxidative stress leads to a buildup of complement component C5a, whereas the complement component C3a remains unaffected. Interestingly, we detected a significant increase in factor H (FH), previously known to inhibit the cleavage of C3 into C3a. Correspondingly, the thrombin levels were significantly increased in the retina of dKO mice, similarly as occurs in aging. These data suggest that C5a was activated via a C3 independent thrombin-mediated C5a process.

## 2. Materials and Methods

### 2.1. Ethics and Animal Experiments

Similar to work performed in our laboratory previously, dry AMD-like *NFE2L2/PGC-1α ^-/-^* [24]. 1-year-old male mice (n = 3, 6 eyes) and sex and age matched wild type controls (n = 3, 6 eyes) were used here. All animals were bred and housed in the Laboratory Animal Centre of University of Eastern Finland, Kuopio. The basic, 3R-principles were implemented in the animal studies.

### 2.2. Genotyping and Tissue Preparation

The global double knockout (dKO) mice were made by knocking down *NFE2L2* and *PGC-1α*. The animals were genotyped using PCR. *NFE2L2* genotypes were detected with 3 primers: LacZ, 5′-GCG GAT TGA CCG TAA TGG CAT AGG; *NFE2L2*–5′, 5′-TGG ACG GGA CTA TTG AAG GCT G; *NFE2L2*–3′, GCC GCC TTT TCA GTA GAT GGA CG. The *PGC-1α* genotypes were determined with 4-primer PCR; WTA, 5′-CCA GTT TCT TCA TTG GTG TG; WTB, 5′-ACC TGT CTT TGC CTA TGA TTC; KOA, 5′-TCC AGT AGG CAG AGA TTT ATG AC; KOB, 5′-CCA ACT GTC TAT AAT TCC AGT TC [24].

Both PCR reactions included 4 μL of DNA extracted from mouse ears, reaction buffer, 100 μM of each dNTP, 1.5 mM of MgCl2, 1 μM of each primer, and 1.2 U of DreamTaq DNA polymerase (Thermo Fisher Scientific Waltham, MA, USA), in a reaction volume of 30 μL. In the *NFE2L2* genotyping, the reaction conditions were as follows: denaturation at 95 °C for 5 min, followed by 35 cycles at 95 °C for 30 s, at 59 °C for 30 s, and at 72 °C for 45 s, and a final extension at 72 °C for 7 min. In the assessment of *PGC-1α*, samples were denatured at 95 °C for 5 min, followed by 38 cycles at 95 °C for 30 s, 58 °C for 30 s, 72 °C for 30 s, and a final extension at 72 °C for 7 min. The amplicon sizes for the wild type allele of *NFE2L2* and *PGC-1α* are 700 bp and 600 bp, respectively, and the KO alleles for both are 400 bp [24].

### 2.3. Immunohistochemical Staining

The tissue sections were deparaffinized using xylene for 10 min and rehydrated using EtoH. Then, the glass sections were incubated for 25 min in the dark with 0.5% Sudan black B (Acros Organics, Branchburg, NJ, USA) in 70% EtOH, followed by 70% EtoH wash. The washed slides were pre-treated according to antibody vendor’s recommendation with Tris-based or citrate-based antigen unmasking solution (Vector laboratories. Inc, Burlingame, CA, USA) for 10 min at 90 °C. The sections were then quenched with 0.1 M glycine in PBS for 10 min followed by a 0.1% Triton-X in 1X TBS wash for 10 min. Quenched slides were incubated with 20% goat serum for 30 min followed by primary antibodies (Table 1) and incubated overnight at 4 °C. Then, the goat anti-rabbit Alexa Fluor 594 (A11037) (ThermoFisher Scientific, Waltham, MA, USA) secondary antibodies diluted at 1:500 were added and incubated for 3 h. Finally, DAPI (Sigma Aldrich, St. Louis, MO, USA) was added and incubated for 30 min followed by 10 min wash with 1X TBS. Finally, the slides were mounted using Mowiol mounting media and stored in the dark at room temperature for further analysis.

### 2.4. Confocal Imaging and Analysis

The fluorescent stained sections were examined under a confocal microscope (Zeiss AX10 Imager A2, Zeiss, Göttingen, DE) using a 20× (EC Plan-Neofluar 20×/0.50 M27) objective. The microscopic settings were kept identical for all pictures taken and held constant during imaging. Representative high power microphotos were taken close to the vicinity of the optic nerve with ZEN blue v2.3 (Carl Zeiss Microscopy, Göttingen, DE). At least 27 images were taken from 9 sections per eye for all markers. Images were color enhanced using Adobe Photoshop^®^ for visual representation. All the captured images were processed using ImageJ v1.52a (https://imagej.nih.gov/ij/, accessed on 3 July 2021). The background was subtracted using a default rolling ball radius method. Regions of interest (ROIs) were drawn followed by mean gray-value measurement. ROIs were kept constant within each antibody analyzed. All the imaging analyses were blind quantified by at least 3 independent researchers.

### 2.5. Statistics

All data are presented as mean ± SEM (standard error of the mean). Mann–Whitney, a nonparametric test, was used to determine the statistical significance in the mean grey-value (signal intensity) analysis. *p* < 0.05 was considered statistically significant. “ns” represents statistical non-significance.

## 3. Results

### 3.1. Elevated TLRs in the dKO Retina

**Figure 2:**
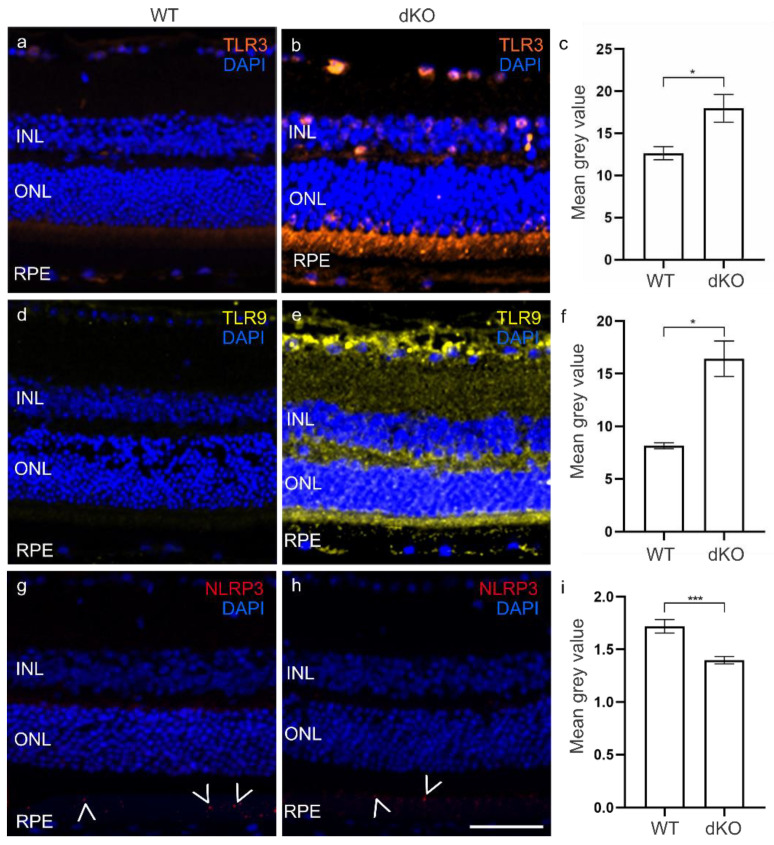
Confocal microscopy analysis of the TLRs and NLRs. One-year-old WT and dKO mice retina focusing on the vicinity of the optic nerve (**a**–**h**). In dKO retina, TLR3 (**b**) and TLR9 (**e**) showed significant increases by ~42%, * *p* = 0.04 (**c**), and ~100%, * *p* = 0.03 (**f**) compared to WT retinas (**a**,**d**), respectively. The dKO retina (**h**) displayed ~19%, *** *p* = 0.0005 (**i**), a significant decrease in NLRP3 levels as compared to WT retina (**g**). Scale = 20 µm.

The oxidative stress-induced reactive oxygen species (ROS) is known to activate DAMP-associated PRRs, which in turn engage downstream signaling pathways producing inflammatory cytokines leading to cellular necrosis [21]. The levels of TLRs, TLR3 and TLR9 were initially assessed in the inner nuclear layer (INL), outer nuclear layer (INL) and RPE layer (Figure 2, Figure S1). In dKOs, we observed a ~42% significant increase in TLR3 levels and ~100% elevation in TLR9 levels compared to WT (Figure 2c,f). The presence of damaged mitochondria and ROS on NLRP3 activation in RPE has been implicated in AMD progression [19]. We further assessed the NLRP3 levels in dKO retina and found that these had significant decreased by ~19% compared to WT RPE cells (Figure 2i, Figure S1).

### 3.2. C3 Independent C5 Activation

**Figure 3:**
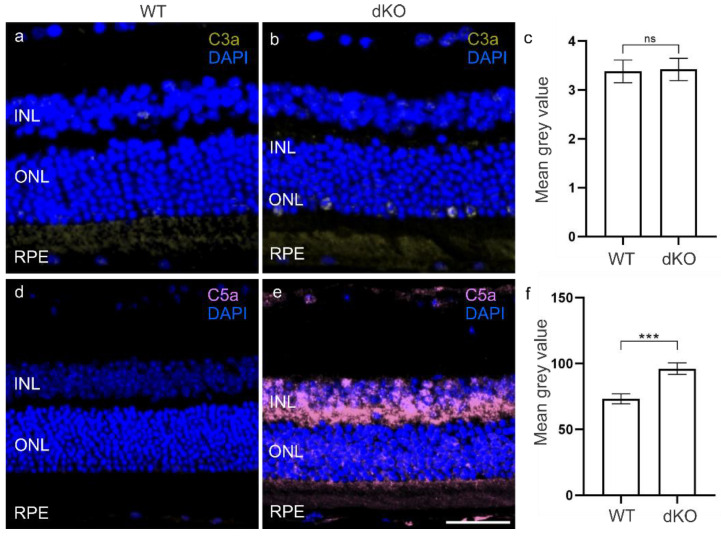
There was no statistical difference in the amounts of the complement component C3a in dKO (**b**) and WT (**a**) retina, *p* = 0.93 (**c**). In contrast, C5a levels were significantly increased in dKO (**e**) by ~30%, *** *p* = 0.0002 (**f**) compared to WT retina (**d**). Scale = 20 µm. ns—not significant.

**Figure 4:**
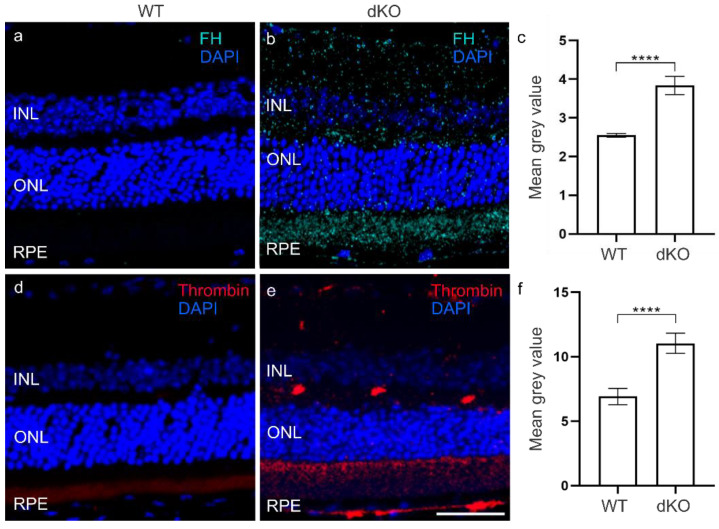
Analysis of levels of complement regulator factor H were significantly elevated by ~50%, **** *p* = 0.0001 (**c**), in dKO (**b**) compared to WT retina (**a**). The thrombin levels were significantly increased by ~60% in dKO retina (**e**), **** *p* = 0.0001 (**f**) compared to WT retina (**d**). Scale = 20 µm.

The CS plays a crucial role in mediating tissue damage and recruiting phagocytes to remove debris and opsonize dead cells to allow their phagocytosis after oxidative stress [11,12]. All three principal CS pathways ultimately end in the generation of its active fragments, notably C3a and C5a, responsible for the recruitment of inflammatory cells. We then analyzed the amounts of the C3a and C5a anaphylatoxins (Figure 3). In dKO retina, we observed no changes in the C3a levels compared to WT retina (Figure 3c, Figure S2). However, interestingly, the C5a levels in the INL, ONL and RPE layer of dKO retina (Figure S2) were significantly increased by ~30% in comparison with WT retina (Figure 3f). Puzzled by this finding, we then analyzed FH, a major inhibitor of the alternative pathway (AP) of the CS [25]. Immunohistochemical analysis of dKO retinas showed a ~50% significant increase in the FH levels compared to WT retina (Figure 4c, Figure S3), i.e., potentially representing a proactive inhibition of C3 cleavage to C3a by FH [25,26]. In the next stage to explain why the C5a level had become elevated (Figure 3f), we analyzed thrombin, an enzyme known to substitute for C3-dependent C5 convertase [27]. Compared to the WT retinal thrombin levels (Figure 4d), we detected a ~60% significant increase (Figure 4f) in the INL, ONL and RPE layer of dKO retina (Figure 4e, Figure S3). These results suggest the presence of an intriguing cross-talk between FH and the CS involving thrombin.

### 3.3. CRP and RAGE Coordinate under Oxidative Stress

**Figure 5:**
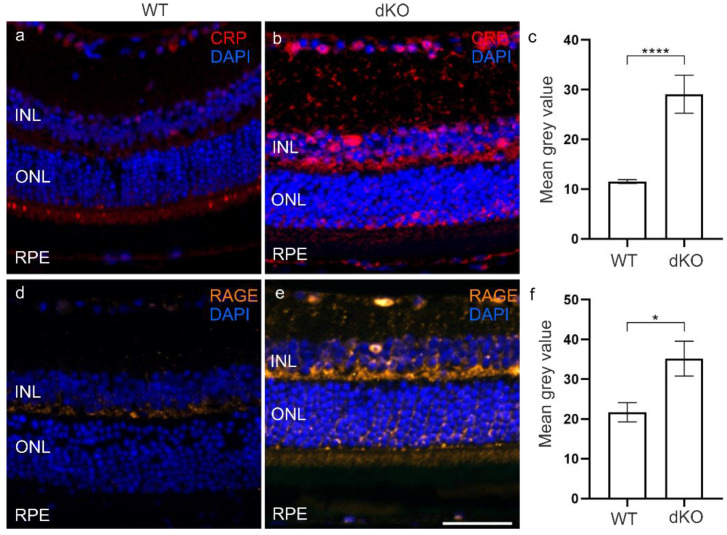
The CRP levels in the dKO retina (**b**,**c**) were significantly increased by ~150%, **** *p* = 0.0001, and RAGE levels by ~60%, * *p* = 0.01 (**e**,**f**), as compared to WT retinas (**a**,**d**). Scale=20 µm.

Oxidative stress and elevated ROS levels have been claimed to increase the CRP level in various diseases [19,28,29,30,31,32]. FH is involved in regulating the CS and known to correlate positively with CRP levels [33,34]. Moreover, CRP is able to inactivate the AP of the CS [35,36]. We analyzed the levels of CRP in both dKO and WT retinas (Figure 5). There was a ~150% significant increase in the CRP level in dKO retina as compared to WT retina in the INL, ONL and RPE layer (Figure 5c, Figure S4). This is in line with studies revealing the contribution of CRP in the host defense system by limiting the damaging effect of late CS as well as its close coordination with FH. Subsequently, CRP has also been shown to stimulate RAGE expression when there are conditions of oxidative stress induced by ROS production. In addition, RAGE acts as a promotor for DAMPs [19,37]. In dKO animals, we observed a ~60% significant increase in the RAGE levels in comparison to WT mainly in the INL, ONL and RPE layer (Figure 5, Figure S4).

Altogether, these results suggest that when there is chronic oxidative stress in the retina, this initiates a proinflammatory response and the activation of the CS, which can be modulated by elevated levels of FH and thrombin in conjunction with those of CRP and RAGE.

## 4. Discussion

Aging-related mitochondrial damage, oxidative stress and ROS production have been shown to be involved in several neurodegenerative diseases, including AMD, Alzheimer’s disease, and Parkinson’s disease [38,39]. Para-inflammation is a process in which specific tissues adopt and respond to various stresses in attempts to maintain homeostasis [40]. With tissue aging, there is a sustained buildup of low-grade chronic inflammation accompanied by oxidative stress, more and more mitochondrial damage, and an accumulation of detrimental oxidized cellular particles [41,42,43]. The mitochondrial derived ROS, mtDNA and oxidized particles can directly induce a non-infective (“sterile”) systemic chronic inflammation (SCI) as these signals are recognized as DAMPs by PRRs, leading to the triggering of intracellular signals resulting in the production of pro-inflammatory cytokines and chemokines [19,44,45,46]. Unlike the adaptive immune response, the innate immunity lacks fine specificity, but it can distinguish non-self PRRs from self-PRRs [47]. Multiple studies have demonstrated the vital role of innate inflammation in the progression of AMD [16,18,19,48,49,50]. Here, we employed our established *NFE2L2/PGC-1α ^-/-^* mouse model mimicking many of the clinical features of dry AMD to clarify the dynamics of the inflammatory response in the retina to oxidative stress and mitochondrial damage.

Although in general TLR3 has been shown to react to double-stranded RNA, emerging studies have revealed that it has a variety of functions, including its role to combat oxidative stress and innate inflammation. TLR3 acts as an amplification regulator of the immune response and serves as an endogenous sensor of necrosis, independent of viral activation [51]. Furthermore, TLR3 activation mediated by signal transducer and activator of transcription 3 (STAT3) significantly increased RPE cell viability in situations of oxidative stress [52]. TLR3 is involved in the regeneration/healing of the central nervous system [53]. The treatment of epithelial cells with H_2_O_2_ increases the expression of TLR3, whereas pre-treatment with the antioxidant N-acetylcysteine (NAC) reverses TLR3 expression [54]. It has also been reported that oxidative stress and pro-inflammatory responses increase TLR3 levels [55,56]. In line with these reports, our immunohistochemical analysis of TLR3 showed a significant increase in dKO retina, supporting the modulatory effect of TLR3 in times of oxidative stress. The release of mtDNA and oxidative stress have long been hypothesized in AMD progression, and recent studies have highlighted their tight regulation with TLR9 signaling and inflammation [57,58,59]. Previously, we showed that elevated oxidative stress results in incomplete mitochondrial repair mechanisms and an accumulation of oxidized undigested mitophagy aggregates [60]. In this study, we found substantial levels of TLR9 in the retinas of our dKO mice. This could potentially be attributable to the presence of oxidized mtDNA fragments in the cytoplasm or the extracellular environment acting as a DAMP to TLR9, and thus as stimulants to inflammation [61]. This suggests that both TLR3 and TLR9 can also respond to local oxidative stress and be associated with distinctive changes in selective TLR expression in the retina, e.g., by initiating an innate immune response. In addition, oxidative stress and TLR9 have been shown to mediate the NLRP3 inflammasome [19,58]. In particular, the activation of the NLRP3 inflammasome has been linked with inflammation and AMD pathogenesis [19,62]. In our dKO retina specimens, NLRP3 was significantly decreased compared with WT sections. Here, we suggest that although oxidative stress and mitochondrial damage were exacerbated in RPE cells, they might tend to switch on TLR activation, which can recruit inflammatory cells to the impaired tissue, leading to the clearance of damaged cells and thereby resolving the inflammation.

Emerging evidence suggests that there is a crucial crosstalk between TLRs and the CS in innate immunity [63,64,65]. Nevertheless, CS activation itself is known to contribute to AMD progression via the AP, as reviewed in detail elsewhere [66,67,68,69]. Our findings show abundant amounts of C5a in dKO retina, whereas C3a levels remained comparable to those present in WT retina. Our working hypothesis was that there would be equally high levels of both C3a and C5a; to clarify this unexpected finding, we then analyzed FH. FH is a known key regulator responsible for switching on the complement cascade by accelerating the decay of C3 convertase and preventing the elimination of healthy cells, but not damaged cells [67,70,71]. The protective role of FH is independent of the membrane attack complex (MAC) [72]. In addition, FH tends to accumulate in drusen, and its role has been discussed in the progression of AMD [70,73,74]. In this study, the observed significant increase in FH might explain the unchanged C3a level found in dKOs [25,26,75]. However, the elevated amount of C5a must be attributable to some different cellular mechanism. Previous studies conducted in RPE cells detected a decrease in FH expression during oxidative stress lasting 48 h [76]. Here, we suggest that in times of sustained chronic oxidative stress, the dKO cells activate some kind of self-defense mechanism, which might overcome the accompanying FH decline. Similarly in our previous studies, we observed a significant increase in autophagy during oxidative stress; this was, however, inadequate to maintain cellular homeostasis [24,60].

**Figure 6:**
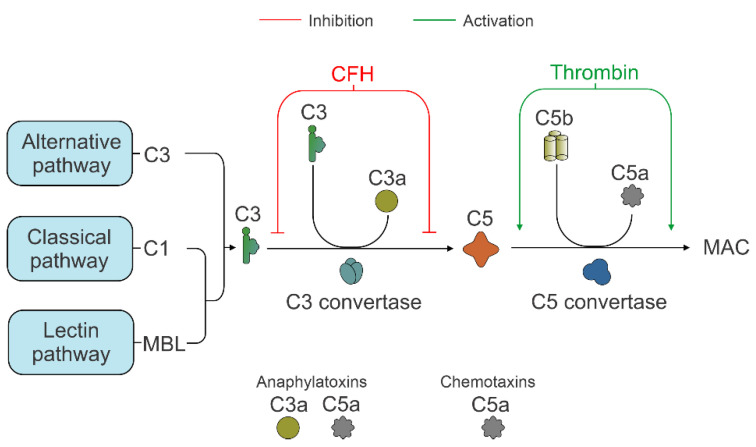
A schematic representation of the complement system in retina of *NFE2L2/PGC-1α* dKO. Under conditions of oxidative stress and mitochondrial damage, there is a state of chronic inflammation involving an inhibition of component C3 via factor H (FH) and an activation of C5a and therefore of the terminal pathway; this is mediated by thrombin. However, further studies will be required to clarify the role of thrombin in chronic inflammation and AMD pathogenesis. Complement components, C1, C3, C3a, C5a and C5b; MBL (mannose-binding lectin); MAC (membrane attack complex).

There are a growing number of studies demonstrating synergy between coagulation and the CS pathways [77,78]. Thrombin, a serine protease, was able to generate biologically active C5a in C3^-/-^ mice, enhancing the terminal pathway of the CS [27]. The FH inhibitory effect of CS was not affected by thrombin, whereas the incubation of FH with the plasma thromboplastin activator, FXIa, reduced the capacity of FH to enhance the cleavage of C3b [25,79]. Accordingly, we observed significantly elevated thrombin levels in our dKO retina specimens, suggesting that C5a activation occurred independent of C3 of the AP (Figure 6). In addition, thrombin has been shown to stimulate RPE cell proliferation by stabilizing cyclin D1 in the G1 phase of the cell cycle [80,81,82,83]. Further studies will be needed to clarify the role of thrombin in the CS and RPE proliferation in AMD.

The major acute phase protein CRP regulates complement activation by binding FH, and this process has been postulated to be involved in AMD progression [35,84,85,86]. Furthermore, a TLR3 deficiency significantly impaired the expressions of both CRP and IL-6 [87]. The levels of TLRs, such as TLR3 and TLR9, have been reported to be linked to CRP expression [88]. Therefore, in our dKO retinal specimens, the increase in CRP levels might involve an FH-mediated tight AP regulation of the CS, preventing host cell destruction in the chronic inflammatory condition. This finding is in line with immunohistochemical analysis of human donor eyes, which have revealed that high levels of CRP and insufficient FH leading to uncontrolled complement activation were associated with cell and tissue damage [89]. It is known that CRP and RAGE mediated inflammation are involved in AMD progression [19,21,90,91,92]. For example, CRP increased RAGE gene expression and attenuated the degradation of RAGE [93,94,95]. Thus, irrespective of whether the elevated level of RAGE in dKO retina is ROS-mediated and/or CRP-mediated, RAGE is known to exert a deleterious effect in AMD.

## 5. Conclusions

Previously, we demonstrated elevated oxidative stress, dysfunctional mitophagy and accumulations of detrimental oxidized materials in the *NFE2L2/PGC-1α* ^-/-^ dKO mouse model. Here, we provide evidence that there is the presence of a sustained and worsening chronic inflammation. The regulation of the inflammatory response, particularly of the complement system by thrombin, provides an intriguing pathway for modulating the progression of AMD. Further investigations with the *NFE2L2/PGC-1α* ^-/-^ dKO mouse model might potentially reveal novel targets for the prevention or treatment of dry AMD. Furthermore, we would argue that *NFE2L2/PGC-1α ^-/-^* dKO mice display many unique characteristics of clinical dry AMD, and perhaps these animals can be exploited for target discovery and drug validation.

## Figures and Tables

**Table 1:** List of primary antibodies.

Primary Antibodies Against	Isotope	Working Dilution	Supplier/Catalogue Number
C5a	Polyclonal	1:250	AB217027
C3a	Monoclonal	1:100	NBP2-66994
FH	Polyclonal	1:50	NBP2-90802
C-reactive protein	Polyclonal	1:100	AB65842
NLRP3	Polyclonal	1:100	AB214185
RAGE	Polyclonal	1:100	AB3611
Thrombin	Monoclonal	1:100	SC271449
TLR3	Polyclonal	1:100	AB62566
TLR9	Polyclonal	1:100	AB37154

## Data Availability

The data supporting the findings of this study are available upon reasonable request.

## Supplementary Materials

The following are available online at https://www.mdpi.com/article/10.3390/biology10070622/s1, Figure S1: The TLR3 levels in INL (*p* < 0.0001), ONL (*p* < 0.0001) and RPE (*p* = 0.04) layers of dKO animals were significantly increased compared to WT animals (**a**–**c**). Similarly, the TLR9 levels were significantly increased in dKO animals INL (*p* = 0.001), ONL (*p* = 0.03) and RPE (*p* < 0.0001) layers (**d**–**f**). However, NLRP3 levels in remained unchanged in INL (*p* = 0.95), followed by non-significant decrease by ~15% in ONL (*p* = 0.19) and non-significant increase by ~8% in RPE (*p* = 0.35) (**g**–**i**), Figure S2: The C3a levels in INL significantly decreased by ~ 40% (*p* = 0.01), followed by non-significant decrease by ~28% in ONL (*p* = 0.05) and non-significant ~61% increase in RPE (*p* = 0. 49) layers of dKO animals compared to WT animals (**a**–**c**). The C5a levels were significantly increased in dKO animals INL (*p* = 0.02), ONL (*p* < 0.001) and RPE (*p* < 0.0001) layers (**d**–**f**), Figure S3: The FH levels in INL (*p* < 0.0001), ONL (*p* < 0.0001) and RPE (*p* < 0.0001) layers of dKO animals were significantly increased compared to WT animals (**a**–**c**). Similarly, the thrombin levels were significantly increased in dKO animals INL (*p* = 0.0001), ONL (*p* = 0.0001) and RPE (*p* < 0.0001) layers (**d**–**f**), Figure S4, The CRP levels were significantly increased in dKO animals INL, ONL and RPE (*p* = 0.0001; *p* = 0.0001; *p* = 0.0001), respectively compared to WT animals (**a**–**c**). The RAGE levels in INL (*p* < 0.0001), ONL (*p* < 0.0001) and RPE (*p* < 0.0001) layers of dKO animals were significantly increased compared to WT animals (**d**–**f**).
