# Supplementary material for: Oxidative Stress and Mitochondrial Damage in Dry Age-Related Macular Degeneration Like NFE2L2/PGC-1α -/- Mouse Model Evoke Complement Component C5a Independent of C3
Source: Biology (Basel). 2021 Jul 4;10(7):622. doi: 10.3390/biology10070622 (PMC8301195; doi:10.3390/biology10070622)
Supplement: Supplementary file 1 [file biology-10-00622-s001.zip › biology-1272841-supplementary.pdf]

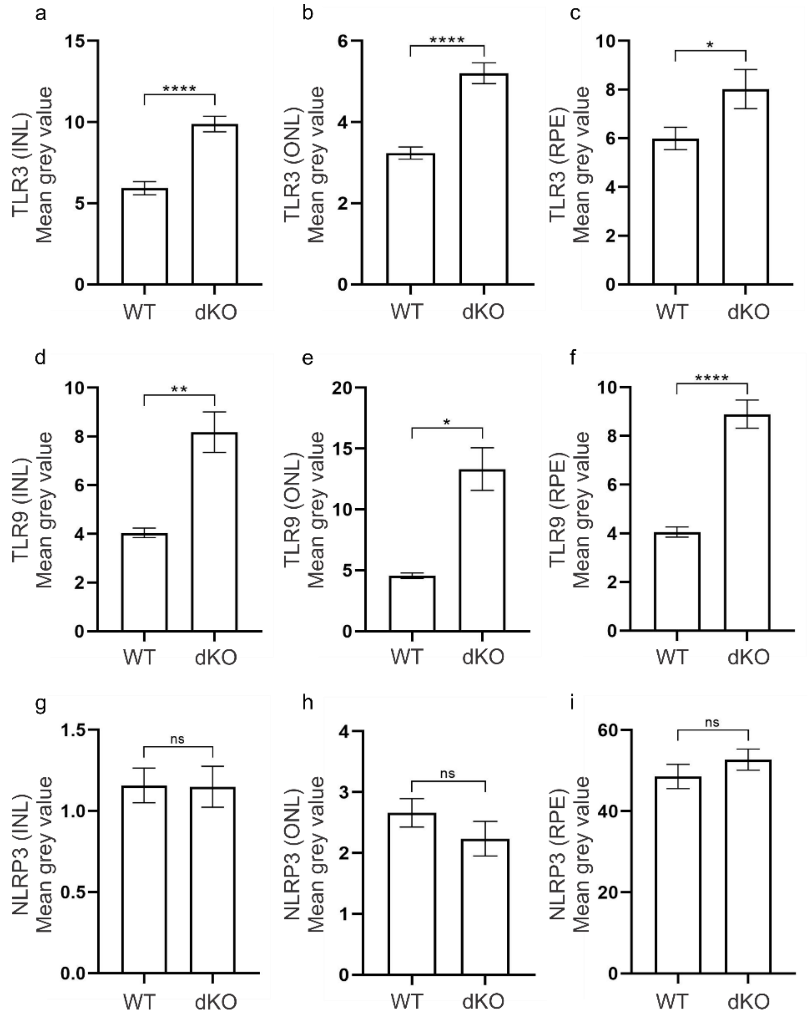


**Figure S1.** The TLR3 levels in INL ($p < 0.0001$), ONL ($p < 0.0001$) and RPE ($p = 0.04$) layers of dKO animals were significantly increased compared to WT animals (**a**, **b** and **c**). Similarly, the TLR9 levels were significantly increased in dKO animals INL ($p = 0.001$), ONL ($p = 0.03$) and RPE ($p < 0.0001$) layers (**d**, **e** and **f**). However, NLRP3 levels in remained unchanged in INL ($p = 0.95$), followed by non-significant decrease by ~15% in ONL ($p = 0.19$) and non-significant increase by ~8% in RPE ($p = 0.35$) (**g**, **h** and **i**). INL (inner nuclear layer); ONL (outer nuclear layer); RPE (retinal epithelial layer).

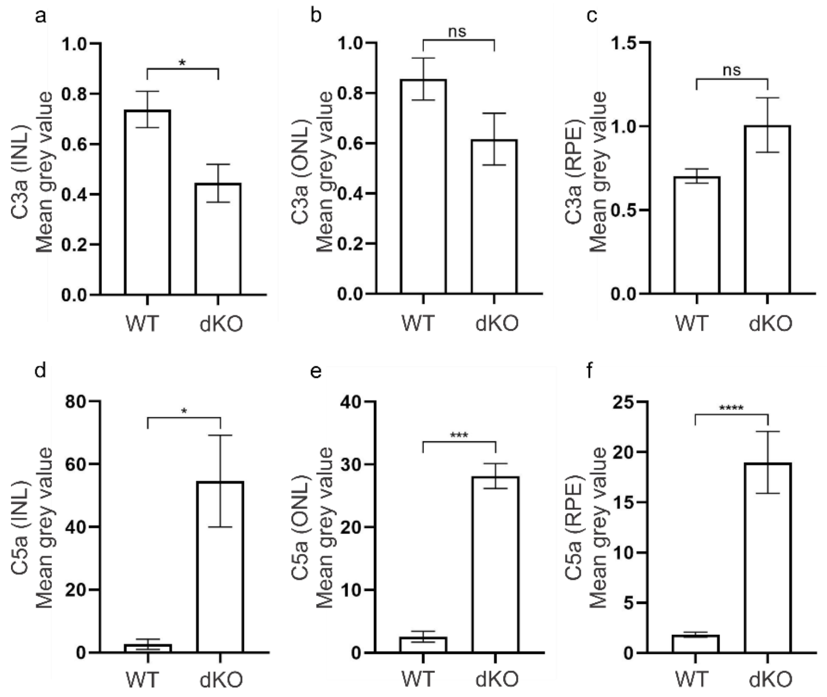


**Figure S2.** The C3a levels in INL significantly decreased by ~ 40% ($p$ = 0.01), followed by non-significant decrease by ~28% in ONL ($p$ = 0.05) and non-significant ~61% increase in RPE ($p$ = 0. 49) layers of dKO animals compared to WT animals (**a**, **b** and **c**). The C5a levels were significantly increased in dKO animals INL ($p$ = 0.02), ONL ($p$ < 0.001) and RPE ($p$ < 0.0001) layers (**d**, **e** and **f**). INL (inner nuclear layer); ONL (outer nuclear layer); RPE (retinal epithelial layer).

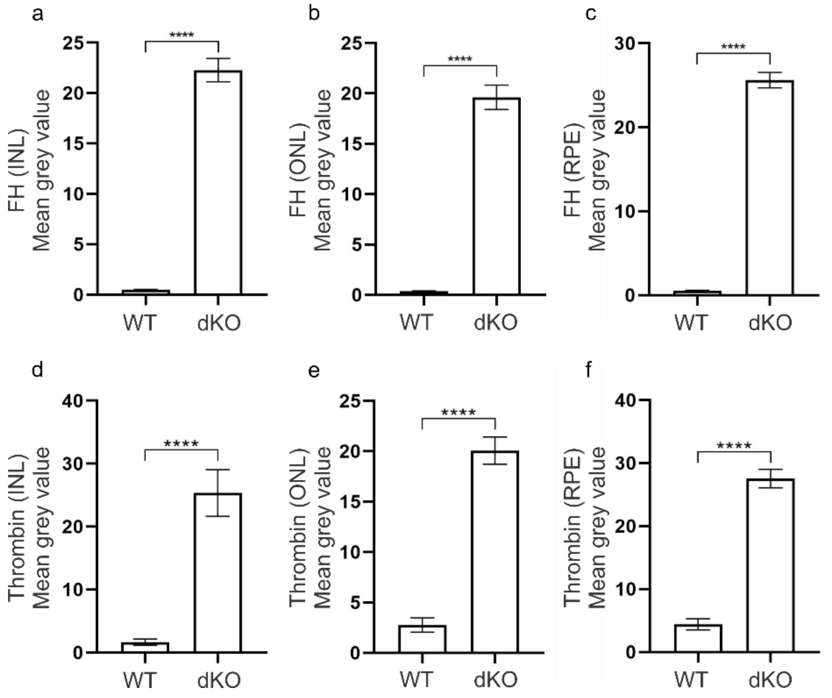


**Figure S3.** The FH levels in INL ($p$ < 0.0001), ONL ($p$ < 0.0001) and RPE ($p$ < 0.0001) layers of dKO animals were significantly increased compared to WT animals (**a**, **b** and **c**). Similarly, the thrombin levels were significantly increased in dKO animals INL ($p$ = 0.0001), ONL ($p$ = 0.0001) and RPE ($p$ < 0.0001) layers (**d**, **e** and **f**). INL (inner nuclear layer); ONL (outer nuclear layer); RPE (retinal epithelial layer).

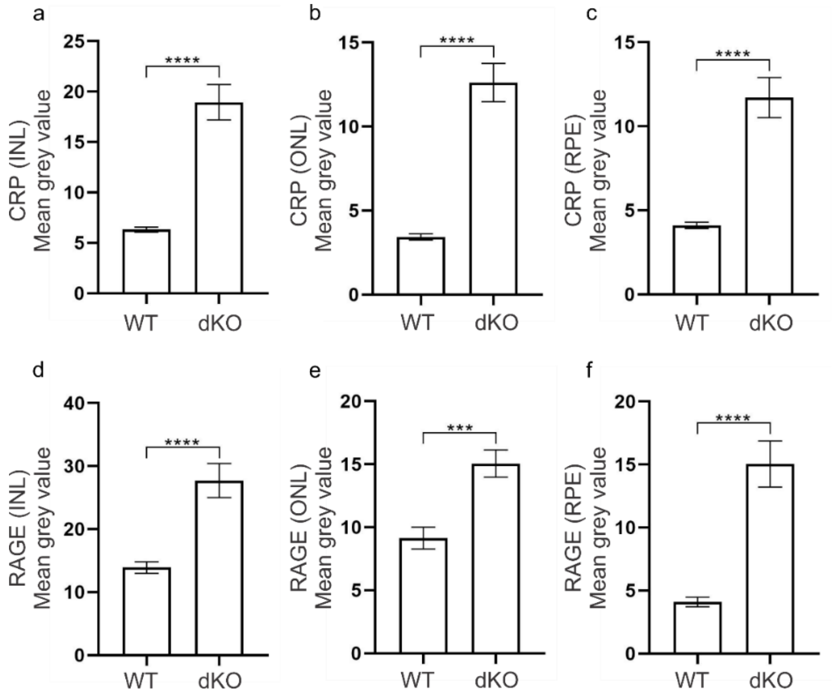


**Figure S4.** The CRP levels were significantly increased in dKO animals INL, ONL and RPE ($p = 0.0001$; $p = 0.0001$; $p = 0.0001$), respectively compared to WT animals (**a**, **b** and **c**). The RAGE levels in INL ($p < 0.0001$), ONL ($p < 0.0001$) and RPE ($p < 0.0001$) layers of dKO animals were significantly increased compared to WT animals (**d**, **e** and **f**). INL (inner nuclear layer); ONL (outer nuclear layer); RPE (retinal epithelial layer).
